# The Voice of Drug Consumers: Online Textual Review Analysis Using Structural Topic Model

**DOI:** 10.3390/ijerph17103648

**Published:** 2020-05-22

**Authors:** Lifeng He, Dongmei Han, Xiaohang Zhou, Zheng Qu

**Affiliations:** 1 School of Information Management and Engineering, Shanghai University of Finance and Economics, No.777 Guoding Road, Shanghai 200433, China; lifenghe819@163.com (L.H.); xiaohang950510@163.com (X.Z.); quzheng90@outlook.com (Z.Q.); 2 Shanghai Key Laboratory of Financial Information Technology, Shanghai University of Finance and Economics, No.777 Guoding Road, Shanghai 200433, China

**Keywords:** online drug review, structural topic model, text mining, consumer concerns

## Abstract

Many web-based pharmaceutical e-commerce platforms allow consumers to post open-ended textual reviews based on their purchase experiences. Understanding the true voice of consumers by analyzing such a large amount of user-generated content is of great significance to pharmaceutical manufacturers and e-commerce websites. The aim of this paper is to automatically extract hidden topics from web-based drug reviews using the structural topic model (STM) to examine consumers’ concerns when they buy drugs online. The STM is a probabilistic extension of Latent Dirichlet Allocation (LDA), which allows the consolidation of document-level covariates. This innovation allows us to capture consumer dissatisfaction along with their dynamics over time. We extract 12 topics, and five of them are negative topics representing consumer dissatisfaction, whose appearances in the negative reviews are substantially higher than those in the positive reviews. We also come to the conclusion that the prevalence of these five negative topics has not decreased over time. Furthermore, our results reveal that the prevalence of price-related topics has decreased significantly in positive reviews, which indicates that low-price strategies are becoming less attractive to customers. To the best of our knowledge, our work is the first study using STM to analyze the unstructured textual data of drug reviews, which enhances the understanding of the aspects of drug consumer concerns and contributes to the research of pharmaceutical e-commerce literature.

## 1. Introduction

In the late 1990s, the first online pharmacy appeared in the United States, selling over-the-counter and prescription drugs. By 2009, there were about 3000 websites selling prescription drugs, and this number had increased to 35,000 in 2016 [1]. At the same time, more and more patients are buying drugs through pharmaceutical e-commerce sites. According to an FDA survey in 2012, 23% of adult online shoppers have bought drugs online [2]. In China, with the rapid progress of internet technology and the change in consumers’ shopping habits, pharmaceutical e-commerce sites have developed rapidly. From 2012 to 2016, the total transaction volume of drug B2C (Business To Customer) business increased by nearly 10 times in China, with a compound annual growth rate of 77.2%, and reached about 28 billion dollars in 2016. With the promotion of pharmaceutical enterprises and e-commerce websites, it is estimated that drug B2C business will maintain a high compound annual growth rate of 41.9% in the future, and will reach about 100 billion dollars in 2026 in China.

The pharmaceutical e-commerce market contains huge commercial value, which not only attracts extensive attention from pharmaceutical companies and e-commerce websites but also arouses the interest of researchers. The prior literature has mainly focused on how to develop pharmaceutical e-commerce. For example, Orizio et al. [3] believed that policy regulations and individual health literacy were two main aspects that affect the promotion of pharmaceutical e-commerce. Roger et al. [4] found that the certification body provided useful information for pharmaceutical e-commerce sites and online consumers, which can help consumers make better purchasing decisions. In addition to macro policy factors, understanding individuals’ evaluation of online pharmacies at the micro level and analyzing consumers’ concerns when they buy drugs online can provide an important decision-making basis for the development and improvement of pharmaceutical e-commerce services [5,6]. By comparing the online price with the offline one, the CIPA survey found that cost saving was paramount for consumers to buy drugs online [5]. Monteith et al. [7] documented that the benefits of shopping for drugs online include convenience and low price. In addition to considering the lower price and availability, Abanmy et al. [6] conducted a more detailed survey and identified four factors for consumers to buy drugs online. They are unavailability in the local market, a cheaper price, convenience, and good services such as home delivery and refill reminders by email. 

At present, most of related research is based on population-level surveys. The data are obtained from questionnaires or interviews. However, questionnaires are time-consuming and laborious in data acquisition, which is an inefficient method [8]. Besides, the quality of the data obtained from surveys depends on the complexity or length of the questionnaire and the willingness of respondents to participate. The subjective bias will make it difficult to replicate the results of these studies [9,10]. Moreover, the data obtained from surveys may quickly become outdated. Therefore, it is worthwhile to consider other alternative sources of data for finding out consumers’ concerns when they buy drugs online.

Online reviews, as an important source of information, provide an alternative solution to alleviate the problem [11]. In China, many pharmaceutical e-commerce sites have launched review mechanisms that allow consumers to post online reviews based on their purchase experiences. Compared with traditional market research methods such as questionnaires, online reviews published on platforms are all public, which can obtain a large number of reviews at a low cost and in a short time [12]. In addition, reviews may better reflect the real opinions of consumers [13], as users participate voluntarily. Furthermore, online reviews are updated in real time, which can enable us to grasp changes in factors in a timely fashion through real-time analysis techniques [14].

In this paper, we intend to examine what consumers say about their experience after buying drugs from online platforms by analyzing a large empirical dataset collected from one of the largest e-commerce sites (JD.com, Company Location: Beijing, China.) in China. These findings can be translated into management insights that can lay the foundation for pharmaceutical e-commerce sites and drug companies to improve customer satisfaction and corporate performance. Specifically, first of all, we apply the structural topic model (STM) to reliably identify consumer concerns about online drug purchases through online reviews. Furthermore, the STM allows us to incorporate document metadata (e.g., whether reviews are positive or negative) into the data generation process of the corpus, resolving the limitations of the traditional Latent Dirichlet Allocation (LDA) [11]. This advantage enables us to study the generation of positive and negative reviews by means of rigorous statistical analysis, thereby identifying negative topics that make consumers dissatisfied, among the consumer concerns [15]. Finally, considering the dynamics and interdependence of the topics, we also analyze changes in them over time.

Our research makes the following two contributions to the literature. The first is our methodological contribution. To the best of our knowledge, we are the first to introduce the STM into online drug reviews, while previous literature was mainly based on the conclusions of questionnaires. Our method helps to reveal and analyze the real voice of consumers who buy drugs online and contributes to the management literature of pharmaceutical e-commerce.

In addition, our research also provides important management practice enlightenment. Based on a large number of review samples, we summarize 12 major topics, three of which have almost not been mentioned in the survey-based existing studies. They are expiration date, after-sale service, and product packaging. Based on the unique advantages of the STM, we reveal consumer dissatisfaction and describe the changing trend of these consumer concerns over time. For the former, we find that the expiration date and after-sales service of the product are the two most important factors that make consumers dissatisfied, which would also have a significant impact on the reputation of the business through the openness of online reviews. However, these are not mentioned in previous studies. For the latter, the prevalence rate of consumer dissatisfaction in the negative reviews shows no obvious downward trend over time, indicating that pharmaceutical e-commerce sites and pharmaceutical enterprises still have huge room for improvement. On the other hand, the prevalence rate of drug price in the prevalence of positive reviews show a declining trend over time, indicating that the low price strategy implemented by pharmaceutical e-commerce and drug companies is gradually losing its effectiveness. On the contrary, customers are paying more attention to the improvement of service quality.

The rest of the study is arranged as follows. In the second part, we review the research literature related to pharmaceutical e-commerce and the topic model. The third part introduces the basic process of data collection and processing. The fourth part describes our data and model setup. The fifth part presents the main results of the study. Finally, the sixth part summarizes our research and analyzes the limitations as well as suggestions for future research.

## 2. Literature Review

### 2.1. Pharmaceutical E-Commerce

Since the 1990s, there has been a flood of literature describing the rapid growth trend of internet applications and the huge potential for market expansion. On this basis, Lowes et al. [16] explained the inevitability of the development of the pharmaceutical industry by virtue of the internet. For the healthcare and medical fields, Gallagher et al. [17] pointed out that the combination of traditional pharmacy and the internet was the only way for enterprises to gain market share. Meanwhile, from the perspective of consumers, it can also improve the experience of obtaining services. This win-win situation perfectly matched the trend of online channel development in the pharmaceutical industry. The combination of information systems and the internet has also brought huge management dividends to medical and health subjects. Some studies have pointed out that the use of hospital information systems can not only significantly improve group members’ satisfaction but also improve the satisfaction of patients, so as to improve the management performance of hospitals [18,19]. Similarly, for the pharmaceutical industry, Ankit et al. compared the sales volume of pharmaceutical e-commerce in 1999 with that in 2010, which had increased from only $1.9 billion to more than $50 billion, with online pharmacies continuing to grow in a spurt in the following years [20]. CSIP [5] reported that more than 35,000 pharmaceutical e-commerce sites have been operating globally as of the end of 2016, after the first online pharmacy was established in the United States in 1999. Pharmaceutical e-commerce sites are also expanding their service areas in the process of development. As described in the Woonsocket et al. report [21], CVS started to try to integrate prescription drug services into its sales system as early as 2004, as this online pharmacy is no longer satisfied with selling only over-the-counter drugs and healthcare drugs.

In view of the broad prospect of pharmaceutical e-commerce, how to promote the development of pharmaceutical e-commerce has become the most important topic for pharmaceutical manufacturers. There has been literature on the impact of macro policy factors on pharmaceutical e-commerce. Macias et al. [22] investigated the quantity and quality of the risk information disclosure of 90 online pharmacies around the world that can supply prescription drugs and found that although most of the sample contents can meet the standards of the FDA, the effect of alternative medical treatment for patients is still open to discussion. Mackey et al. [23] discussed the problem of drug abuse caused by the lax prescription review of online pharmacies and proposed regulatory measures for online pharmacies to ensure public safety. Through a purchase experiment for prescription drugs with 98 pharmaceutical e-commerce sites in Italy as samples, Gelatti et al. [24] found that the general safety of consumers could not be generally guaranteed due to the insufficient strict supervision of pharmaceutical e-commerce, poor drug traceability and poor product quality. After reviewing a series of laws, regulations and corresponding policies to combat illegal online pharmacies in China over the years, Huang et al. [25] illustrated that the security of online pharmacies was a hot issue of global concern and that a considerable number of countries were making efforts to this end.

At the micro level, it is necessary to understand the evaluation of individual consumers on pharmaceutical e-commerce, so as to analyze what concerns customers when they buy drugs online [3,6]. Gallagher et al. [17] argued that consumers’ acceptance of online pharmacies was high due to low enough prices, near-zero service access barriers and sufficient anonymity. However, the privacy leakage brought by technology, the lack of completeness of prescription inspection, the drug storage behind the network and other issues were worthy of concern. Therefore, although the development prospects of pharmaceutical e-commerce are beyond doubt, corresponding regulatory measures should be taken simultaneously to ensure the healthy and sustainable development of the medical system. Fung et al. [26] also believed that the benefits of pharmaceutical e-commerce include reduced product costs and transaction costs, improved anonymity and enhanced convenience, while security should be firmly controlled in the development process. Ankit et al. [20] hold a similar view that providing convenient, fast and cost-effective services was the basis for the rapid development of pharmaceutical e-commerce. According to Van et al. [27], how to build a transportation system with high product exposure, high information comprehensibility, high privacy protection, low cost and fast logistics and to obtain competitive prices was the key to market competition for operators. In other words, the key to the operation of pharmaceutical e-commerce lay in the technology itself. Rajagopal et al. [28] also agreed that the advantage of an online pharmacy was that it can provide faster service at a cheaper price than a physical pharmacy. Meanwhile, the availability of convenience to consumers should be guaranteed so that online pharmacies can retain their advantages and achieve greater development, because convenience is one of the key competencies for the rapid development of online pharmacies. Similar views have been expressed by scholars such as Chordiya et al., Andras et al. and Sugiura et al. [29,30,31]. Konstantinos et al. [32] found that the usability level of websites played an important role in purchasing behavior, which was observed by analyzing Greek consumers’ views on online pharmacies and the motivations of consumption behavior through questionnaires. Ahmad et al. [33] indicated that the readability level and content quality of the available information were increasingly valued by consumers, with the increasing reliance on online medical services in daily life, which was observed by analyzing the consulting information readability level of pharmaceutical e-commerce.

Based on previous literature, understanding what concerns customers when they buy drugs online is a worthy topic for both drug manufacturers and e-commerce sites. However, at present, relevant literature is all based on the questionnaire survey method, which has irremediable shortcomings such as being time-consuming and laborious, having large errors and being difficult to track [8,34]. As a new data source, online reviews can well make up for the shortage of questionnaire surveys [10,13]. Mining product or service reviews have been quite common and successful in the marketing research or management science area, such as using hotel reviews to analyze customer concerns including satisfaction and dissatisfaction [11,15]. However, to our knowledge, no study has focused on using text-mining techniques to examine and analyze drug reviews. Therefore, mining customer concerns from online drug reviews is worthwhile and fills gaps in the existing literature.

### 2.2. Online Reviews and Topic Model

Customer concerns including satisfaction and dissatisfaction are the basis for the positioning, design and improvement of products and services [35]. Satisfaction and dissatisfaction can be affected by either the service failure of providers or the emotions and psychological state of customers. Given that the latter is beyond providers’ control, an essential step is to determine the factors that trigger satisfaction and dissatisfaction. In earlier literature, questionnaire surveys and interviews were popular, which not only took a lot of effort to obtain data but also had to be questioned regarding the accuracy of the survey results [36,37]. With the developing of e-commerce, online users express their demands and suggestions for products and services in the form of online reviews. In this way, online reviews contain a wealth of information, including users’ praise for satisfaction and complaints about dissatisfaction in products and services [38]. Therefore, in recent years, more and more studies have been conducted to analyze the influencing factors for customer satisfaction and dissatisfaction through the text content of online reviews, which are widely applied in the manufacturing and service industries, especially aviation and hotel services [39,40,41].

Before the breakthrough and popularization of natural language processing technology, manual coding was the preferred method to identify factors affecting customer satisfaction from text content. After manually designing a content dictionary, Gebauer et al. [42] analyzed the content of online reviews and found that four factors, including functionality, portability, performance and feasibility, significantly affected customer satisfaction. Levy et al. [9] developed a complaint framework and coded the content of the hotel’s online reviews, finding that room size and staff service were important factors that contributed to customer dissatisfaction. Although manual coding can be coded according to the pre-built framework to ensure the strong explanatory power of the model, manual labeling requires a lot of time and labor, and the reliability of the results is also affected by the initial framework and individual characteristics of the coder [9,10]. The automated text analysis method has become the mainstream method to analyze customer satisfaction factors from text reviews with the development and popularization of natural language technology [43,44], among which the topic model is one of the most widely used methods [45,46,47]. Different from word frequency analysis, LDA is a topic model based on co-occurrence relationships, which outputs topics (word sets with high co-occurrence probability) rather than independent words [48]. For example, Ibrahim et al. [47] used LDA to analyze the relevant tweets collected from the top five online retailers in the UK from Black Friday to Christmas and New Year sales, and found that delivery, products and customer service were the most discussed topics on Twitter.

Studies have also introduced the LDA method to the medical and public health fields [49,50]. Xiao et al. [51] introduced three models similar to LDA and found that studies based on the LDA method can successfully reveal the probabilistic patterns among subjects of adverse drug reactions (ADR), so that ADR can be predicted from a large number of ADR candidates with higher accuracy to obtain drug targets. Zhang et al. [52] adopted the LDA method to extract the topic of doctor characteristics and analyzed the document similarity, so as to further construct iDoctor, a new medical recommendation system, which can significantly improve the accuracy of health advice. The results of over 1.5 million public health tweets analyzed by Paul et al. [53] based on ATAM, a disease-related topic model they proposed, indicated that more detailed and comprehensive public health information can be obtained from social media such as Twitter. Through the textual analysis of doctors’ text feedback, James et al. [54] found that process quality satisfaction was an important driver of patients’ perceived quality while clinical quality better reflected doctors’ perceived quality. Botsis et al. [55] developed a text mining system to extract key clinical features from the vaccine adverse event reporting system (VAERS) narrative, which can help automate the reviewing of adverse event reports. Hao et al. [56] found that doctors’ experience, doctors’ technical skills and attitudes toward patients were the most important factors for patients by modeling the topics of doctors’ online reviews. As these papers demonstrated, automated text analysis has been applied to biometrics, medical referrals and physician evaluations. However, to our knowledge, no study has yet used automated text analysis to study online drug reviews.

Although LDA is widely used as the most popular topic modeling method, it still has the limitation of not being able to model topic prevalence using document-level covariates [11,15]. A significant advantage of the STM compared with LDA is that it allows the connection of arbitrary information, in the form of covariates, with the degree of association of a document with a topic (topic prevalence) as well as the degree of association of a word with a topic (content prevalence) [11]. The STM has become a more suitable method for online reviews because it allows researchers to model topic prevalence using other covariates, such as linking reviews’ extremities to topics and analyzing customer dissatisfaction [12]. In recent years, the STM has been developing in research, in which Roberts et al. [57] analyzed open-ended survey responses in political science; Light et al. [58] focused on the dynamics of the critic valuation of music albums; most recently, Kuhn et al. [59] elaborated on the discourse elements in accident investigation reports. Therefore, our study comprehensively analyzes customer concerns when they purchase drugs online based on the advantages of the STM.

### 2.3. Structural Topic Model

**Figure 1:**
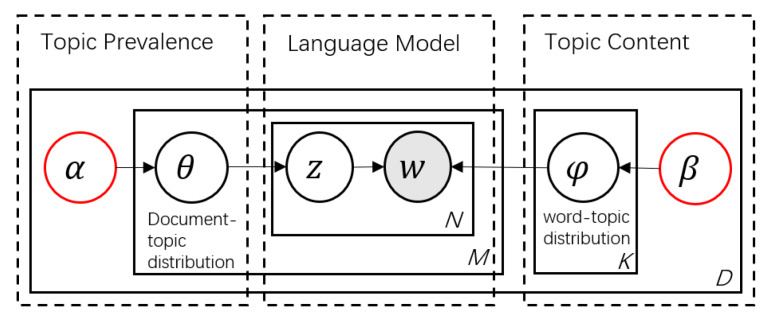
Plate diagram of Latent Dirichlet Allocation (LDA).

The same as LDA, the STM belongs to the topic model of Bayesian generation, which assumes that each topic is a distribution of words and each document is a mixture of topics within the scope of the corpus [60]. LDA was proposed by Blei et al. [48] in 2003. Given the use of an efficient probabilistic inference algorithm to process large scale data, LDA has become a hot method in the text field such as Twitter and blogs. Figure 1 presents the model diagram of LDA. The rectangles denote replication: n∈{1,2,…,n} represents the word in the index document; k∈{1,2,…,k} is indexed for each topic that is user-specified; and d∈{1,2,…,d} represents the document index. The hidden and observation variates are shown by unshaded nodes and the shaded node, in which only the word *w* in the text can be observed. The word distribution of the topic is φ, which means P(w|z) subject to the parameter β for the Dirichlet distribution. The topic distribution of the document θ obeys a Dirichlet distribution with parameter α. The LDA model reflects the text into the potential topic space so that the implied topic in the text can be mined and the probability distribution of the document-topic (topic-word) can be obtained, which is conducive to the further analysis of the text and topic [34].

The model of LDA assumes the following generative process for each document d in the corpus [49,60]:

- Randomly choose a topic distribution $\theta_{d}$ for the document *d*;
- From the distribution of topic $\theta_{d}$, randomly choose a topic $z_{d,n}$;
- Randomly choose a word $w_{n}$ from the corresponding distribution over the vocabulary $\beta_{k,d}$, where $k=z_{d,n}$;
- Return to Step 2 and iterate over all the words $w_{n}$ in the document *d*.

**Figure 2:**
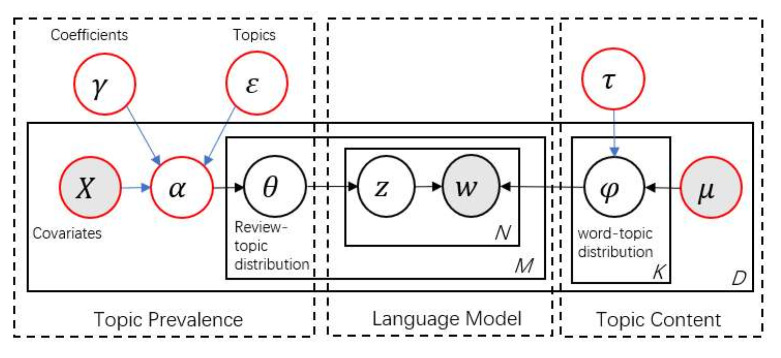
Plate diagram of structural topic model (STM).

In the LDA model, both the document-topic distribution and topic-word distribution are determined by the Dirichlet distribution of the data on the basis of the preset parameters. In reality, the text has the characteristics of heterogeneity (e.g., online reviews). The emotion and information of the text also affect the document-topic and topic-word distributions of the document. Figure 2 plots the model diagram of the STM. Similar to LDA, the STM is also composed of topic prevalence, language model and topic content. In terms of model generation, both of them estimate the document topic matrix and topic word distribution matrix according to the observed parameters. The difference is that the significant improvement of the STM is to introduce document-level structure information to affect the topic prevalence (i.e., per document topic ratio) and topic content, thus we can emphasize the research on how document-level covariates affect the applicability of text content. To be specific, different from the LDA model, which shares certain prior Dirichlet parameters α,β, the STM is limited with prior structure information in the form of generalized linear models parameterized by document-specific covariates X,μ [15].

As for the different comparison between LDA and the STM algorithms, it is theoretically proved that the STM is more suitable for our study. At the text type level, document-topic proportions can vary with things like the extremity of the review, the length of the review and the amount of information in the review. In the LDA model, document-topic is a Dirichlet distribution based on a parameter of α. However, in the STM, researchers can change the topic prevalence parameters by adding covariates to affect the topic proportion of the document [15]. Thus, we can easily find how document-topic proportions vary with covariates (e.g., whether reviews are positive or negative). Furthermore, we can also study how other variables (e.g., the time of the review) moderate the effects of covariates of interest by introducing interaction terms of them.

## 3. Data Collection and Processing

The data collection and preparation steps adopted in this study are as follows. Firstly, for obtaining the research data, the online drug reviews come from JD.COM, one of the largest B2C e-commerce platforms in China, which has a large number of review data that can be freely obtained by users. Customers can post not only textual reviews but also digital online ratings after purchasing drugs on JD.com. In addition, not limited to recent time series review data, online reviews posted by customers a few years ago can also be queried. All of these provide convenience for our research. This site has also served as a data source for many previous studies [61,62,63]. We write programs in Python to collect online drug reviews from customers posted on JD.com. The data for building the topic model should cover different hierarchies, such as different brands, different products/services or different categories [14,15]. Therefore, we crawl a total of 79,328 online reviews of 100 kinds of drugs covering 10 major drug categories on the JD.com pharmaceutical e-commerce module. The items include the text content of reviews, the online ratings and the dates of the reviews. The review dates range from 2016 to 2019. The pharmaceutical e-commerce business on JD.com started in 2016, and the number of reviews in 2016 was too small (less than 0.1%), so we only keep the review data from 2017 to 2019. Finally, we remove duplicate samples and get 78,732 online reviews through the above processes.

The second step is to select our research sample from all the available online reviews. Due to the existence of individual selection bias [64] in consumer reviews, online reviews on e-commerce platforms (such as TripAdvisor.com, Amazon.com and ebay.com) follow a positively skewed J-shaped distribution. The shopping process and review mechanisms on JD.com are similar to those on the above websites, so the reviews on the JD.com website also show a positive J-shaped distribution [61,62,63]. In our sample, the percentages of 5-point and 4-point ratings are 79.9% and 5.3%, and those of 1-point ratings and 2-point ratings are 9.3% and 2.8%, respectively. In the previous research on e-commerce platform reputation, reviews with 1- or 2-point ratings are defined as negative reviews, whereas those with 4- or 5-point ratings are positive reviews [15,65,66]. We can find that the number of positive reviews remains overwhelmingly larger than that of negative ones in our sample. In the construction of the STM model, the sample size corresponding to the covariates should be as consistent as possible [15,67,68]. We aim not only to extract topics from drug reviews but also to identify topics that appear significantly more in negative reviews than in positive ones. Therefore, when using review extremities as covariates in the STM model, it is essential to initially filter the positive and negative reviews to balance the sample size of positive and negative reviews [15], which can help us to identify topics that appear significantly more in negative reviews than in positive ones more reliably [15,57].

To alleviate the possible undesirable effects of an unbalanced sample [69], we use the sample selection method, the same as Hu et al., having a similar background and needs in our study [15]. We intend to build a corpus, in which the number of positive and negative reviews is equal. We first define 1-point and 2-point ratings as negative reviews and only 5-point as positive reviews to address the serious imbalance between positive and negative online reviews. Then, we randomly select the positive online review samples with the same number of negative ones from the samples after the above processing step. During the random sampling process, we also set up an equal number of positive and negative reviews for each class of drugs. Finally, we get a total of 19,054 reviews, which would be used to build the topic model. 

The last step is text preprocessing. For Chinese text, a necessary step is word segmentation, which is different from that of English text. We complete this step by using the Jieba package in Python. Jieba word segmentation adopts an algorithm based on Trie Tree Structure, which can efficiently realize word graph scanning and obtain all the possible word formations of Chinese characters in sentences by using word graph scanning. All of these word-forming possibilities form Directed Acyclic Graphs for quick lookups. Jieba is one of the most effective word segmentation tools in the Chinese language (for more information, see https://github.com/fxsjy/jieba). Then, we remove numbers, punctuation marks and stop words including those from standard stop word lists and user-defined word lists such as drug and nice. The above step is also completed in Python.

## 4. STM Model Setup

### 4.1. Topical Prevalence Parameter

Our research target is not only to extract customer concerns from online drug reviews but also to further analyze what concerns can make consumers satisfied or dissatisfied and explore their trends over time. Therefore, we should model how review extremity (positive or negative), review time and their interaction affect the topical prevalence parameter μ of the STM [11,15,58]. The unique advantage of the STM is that it can model how the document-level covariates affect the topical prevalence parameter μ with a generalize linear model [15]. We define two covariates, namely, extremity and time, to represent the review extremity and posting time of the reviews, respectively. The extremity equals 1 if the reviews are negative (with 1- or 2-point ratings) and 0, otherwise (reviews with 5-point ratings). Time is a number that ranges from 2017 to 2019. Since our samples contained different categories of drugs, which might lead to heterogeneity of the review contents, we also incorporate the drug category into the STM and find that it has no significant impact on the document-topic distribution. Therefore, Equation (1) is set in our study to represent the relationship between topic prevalence and the above two covariates in the STM [11,15].

(1) Prevalence=g(Extremity, Time, Review extremity×Time)

### 4.2. Number of Topics

Our research is accomplished by using the STM package in the R language [68]. The basic STM believes that each document (each document corresponds to a review) consists of K topics with a vocabulary of size (V). The number of topics, K is one of the most important parameters of the STM, which needs to be given by researchers. Moreover, in practical applications, the value of *K* is determined to achieve a substantive interpretation of the outcomes rather than for the maximization of the fit [14,15]. The coherence of the words to each topic and exclusivity of the topic are two criteria for the interpretation power of the model outcomes [15]. Consistency indicates that keywords with high probability in a topic may appear simultaneously in the document. Meanwhile, the exclusivity of topic words means that keywords with high probability will not appear in different topics at the same time. In addition, the determination of the quantity K of topics also depends on the intrinsic nature of the corpus. The number of topics in a work is not large in the existing studies on the corpus of online reviews by using the topic model because of the relative homogeneity among the corpus of online reviews [11,14,15,70]. After checking the coherence and exclusivity of the outcomes of a series of models with different K, we select the 12-topic model because this setting can yield the most semantically coherent and exclusive outcomes.

## 5. Results and Analysis

### 5.1. Topic Summary

We built the STM model and show our results in Table 1 based on online drug reviews through the above steps. Each row in Table 1 represents a topic that reflects consumer concerns. The first column represents the label of each topic, which is marked by researchers [14,56,71]. The second and third columns are the outputs of the model. The second indicates the proportion of each topic, which represents the probability of the topic appearing in the corpus. The third lists the seven most important keywords for each topic, corresponding to words of the highest probability in this topic while having a lower probability in other topics. We can get labels for each topic and distinguish one topic from the others by summarizing these keywords. 

The important inferential task is to infer the topic labels by analyzing the top key words [72]. First, we invited three experts to participate in the topic marking including two doctoral candidates majoring in e-commerce and one doctoral candidate majoring in public health management. Second, each expert labeled topics, referring to the keywords per topic and representative reviews. Third, the topics were discussed and voted on until all the experts reached a unanimous decision on the most suitable names for each of the 12 topics. Fourth, we invited two professors of related disciplines to review our results and determine the final result. The effectiveness of the topic model has been proven mathematically and algorithmically [49,57], while there are other studies empirically proving that topic models can effectively extract topics from online reviews [15,56]. However, our study is the first one to apply the STM to drug reviews. Therefore, we compare the topics extracted by the STM model with those obtained by questionnaire statistics, finding that 9 out of 12 topics have appeared in previous literature [3,5,6,7]. Furthermore, we illustrate the advantages of the topic model in expanding new topics, as there are three topics that have almost not appeared in previous literature including after-sales service, product packaging and the expiration date of drugs [3,5,6,7].

### 5.2. Negative Topic Identification and Analysis

Pharmaceutical websites and enterprises may be more interested in consumer dissatisfaction, as consumer complaints expressed through online reviews are more credible and influential [73,74]. Therefore, managers hope to understand the causes of customer dissatisfaction, so as to improve consumer satisfaction and reduce the adverse effects of consumer complaints. Therefore, we add the extremity of reviews as a covariate when constructing the STM model to estimate how it affects the probability of the topic distribution. If the proportion of a topic in the negative reviews is significantly larger than it in the positive reviews, such a topic can be identified as a negative one [11,15]. 

**Figure 3:**
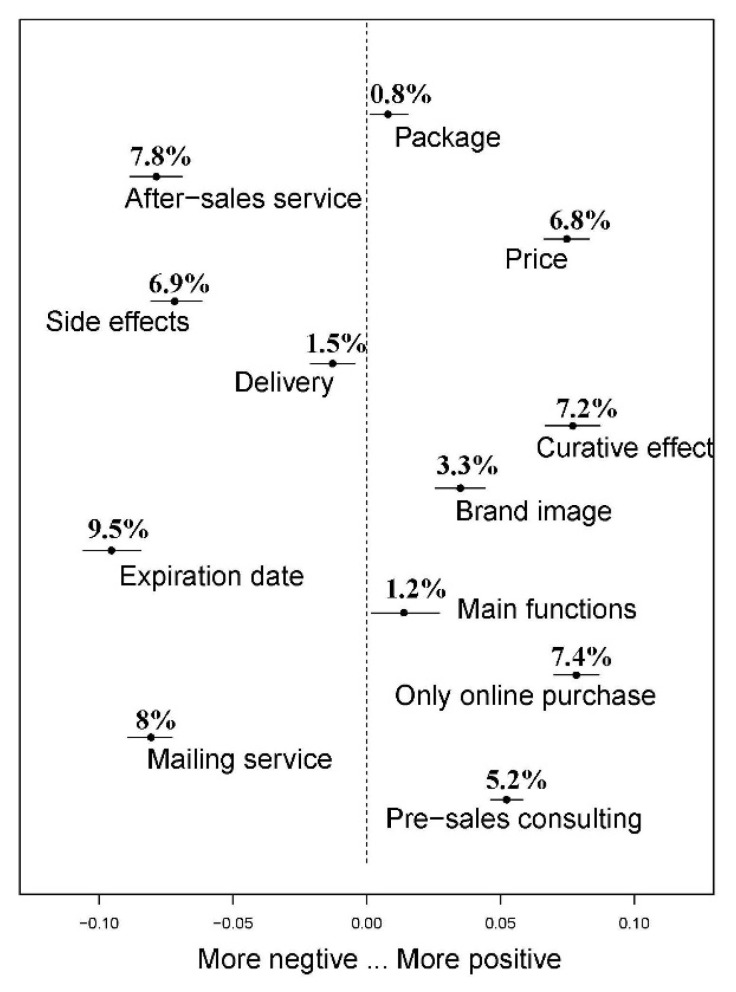
Change in topic prevalence based on review extremity (positive vs. negative).

Figure 3 plots the marginal effect of review extremity on the distribution of the topics, where the points represent the mean values of the estimated differences and the bars represent the 95% confidence intervals of the differences. The longer the distance between the topic and the dotted line (the dotted line indicates zero effect), the more obvious the change in the proportion of the topic in the positive and negative reviews [11]. If we use “price of drugs” as an example, then the proportion of positive reviews is higher than that of negative reviews by 6.8%, while this difference exists in 6.46–7.14% confidence interval. We consider the price of drugs as a positive topic, since the above results indicate that drug price is statistically more likely to appear in positive reviews than in negative reviews. At the same time, topics related to sales services include only online purchase (Topic 10) and pre-sale consulting services (Topic 12), those related to the product itself include curative effect (Topic 6) and main functions (Topic 9), and those related to express delivery include package (Topic 1), and all are positive topics, which is consistent with the previous research [5,6,7].

As mentioned above, we pay more attention to consumer dissatisfaction including after-sales service, the side effects of drugs, distribution services, expiration date and express delivery services. Among them, the expiration date (Topic 8) is the most important factor causing consumer dissatisfaction; what follows are the important reasons for this result. Firstly, differently from in offline physical stores, consumers cannot clearly know the production date of drugs when they buy drugs online. Only when the package is delivered can consumers know the production date of the product, which deprives the right of the consumer to choose drugs produced by manufacturers recently. Secondly, an important purpose of selling products online is to clean up inventory. Merchants give priority to sell products with a long production date, which results in a large number of relevant descriptions of product shelf life in negative reviews and damages the reputation of the merchants. However, we do realize that the expiration date (Topic 8) has almost not been mentioned in previous literature. After-sales service (Topic 2) is also a negative topic. Although previous literature [6,7] has emphasized the role of pre-sale consultation for customers choosing to buy medicines online, it ignores the role of after-sales service. By contrast, our study shows that the after-sales service policies of pharmaceutical e-commerce websites and pharmaceutical companies are not completed, which will lead to consumer dissatisfaction. Topics related to express delivery, including mailing services (Topic 5) and delivery services (Topic 11) are also negative ones. Drugs have stronger timeliness and privacy requirements than ordinary goods, which require faster and safer express delivery services to guarantee. Our study provides a management insight that pharmaceutical e-commerce websites and pharmaceutical enterprises should provide safer, faster and more convenient courier services for online pharmaceutical sales than for ordinary goods, so as to reduce consumer complaints to a certain extent.

### 5.3. Temporal Variations in Costumer Concerns

Customer satisfaction and dissatisfaction constantly originate from whether service providers meet the expectations of customers. Given the changes in user tastes and improvements in enterprise services [75], there are some factors that affect how the satisfaction of drug purchases online will change over time. Therefore, through horizontal comparison, no matter the selection of drugs sold or the improvement of different services, by tracking the importance of customer (dis)satisfaction factors of the business activities over time, the pharmaceutical e-commerce websites and enterprises can adjust their dynamic operation strategies at any time. In this way, not only can customers be catered to, but also pharmaceutical e-commerce websites and enterprises can maximize profits.

**Figure 4:**
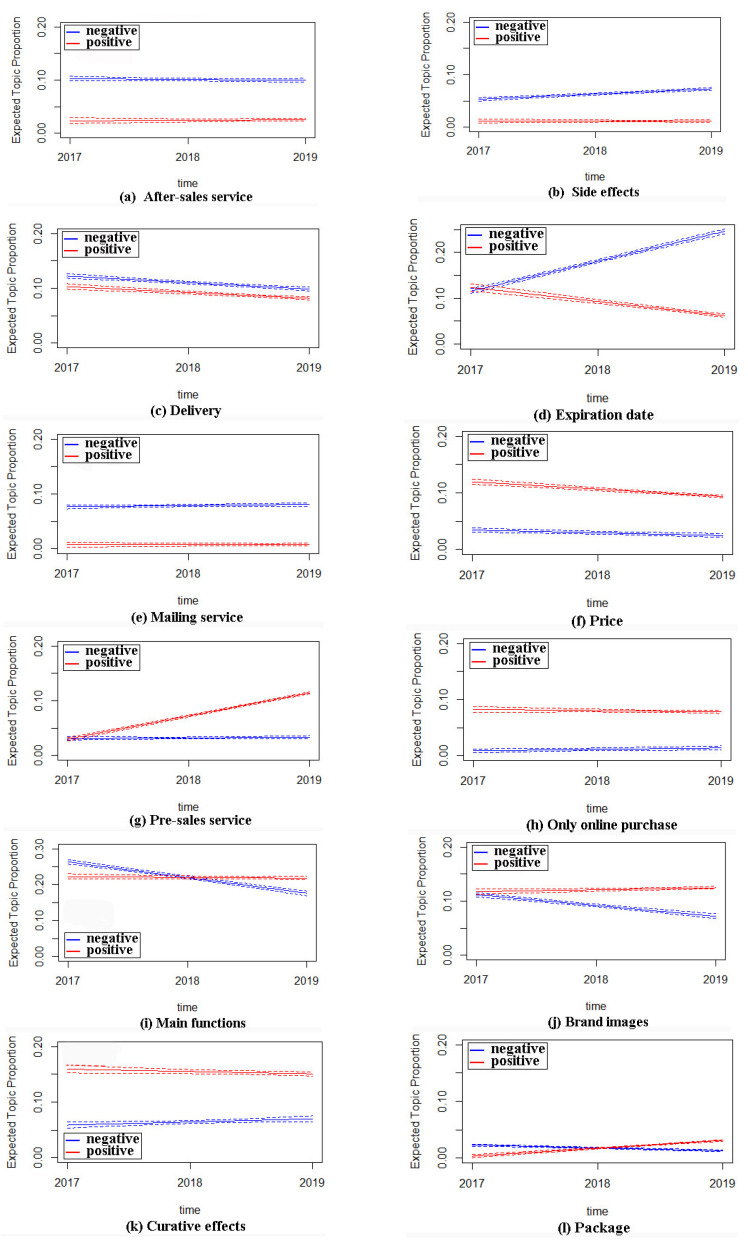
Moderating effects of time.

Studies based on online reviews can better obtain trends of costumer concerns over time compared to questionnaire surveys [11]. Therefore, we implement an interaction term of time and review extremity into the STM model to explore the trend of positive topics (consumer satisfaction) and negative topics (consumer dissatisfaction) over time. We use years as the unit of time, which is just like in the classic research on customer satisfaction over time [14,75,76]. Figure 4 plots the trend of the topic proportion over time with our collected online review data covering the three years of 2017, 2018 and 2019, where the *x*-axis represents time (2017, 2018, 2019) and the *y*-axis represents the topic proportion. The blue and red lines indicate the expected topical proportions of negative and positive reviews. In addition, the dashed line represents the 95% confidence interval for the topic proportion estimates. 

As described above, we focus on five negative topics that change over time. Figure 4a–e show the trend of negative topic proportions over time. Except for delivery service, none of these negative topics shows a downward trend (up or constant) in negative reviews over time. Therefore, pharmaceutical e-commerce websites and enterprises still have great room in the process of improving consumer satisfaction.

From Figure 4b,d, we can find that their prevalence in negative reviews presents an upward trend over time. In particular, the prevalence of the expiration date of drugs (Topic 8) in negative reviews increases from 13% (2017) to 23% (2019), indicating that the expiration date (Topic 8) is gradually becoming the most important element of customer complaints. This also provides an important management inspiration for pharmaceutical e-commerce websites and pharmaceutical enterprises that they should try to maintain the consistency of online and offline medicine production dates after balancing the benefits of clearing stock and the damage of complaints. At the same time, it is important to note that there is an obvious decline in the prevalence of this topic in positive reviews. This phenomenon shows that pharmaceutical e-commerce websites and enterprises should pay attention to the selection of drug categories or raise the access threshold for designated drugs in their later business activities, whether due to the choice of the commodities of e-commerce enterprises or the change in consumers’ value factors.

Figure 4c shows that the topic prevalence of delivery services (Topic 5) in the negative reviews has declined over time, indicating that the level of delivery services has been improved in those years and customers are complaining less and less about the topic. Thus, the pharmaceutical e-commerce websites and enterprises should maintain this level in future drug selling.

From Figure 4a,e we can find that the topic prevalence of after-sales service (Topic 2) and mail service (Topic 11) in positive and negative reviews has not changed significantly over time, which indicates that pharmaceutical e-commerce websites and pharmaceutical enterprises may not sufficiently realize the importance of after-sales service and mail service in improving customer satisfaction and their own credibility, or that they did not put enough effort into improving both. 

Similarly, we can draw conclusions about the trend of positive topic proportions. Figure 4f shows that the topic prevalence of price (Topic 3) in positive reviews has shown a downward trend over time, indicating that the low-price strategies implemented by pharmaceutical e-commerce websites and enterprises have become less attractive to customers. Of course, consumers are always more willing to accept low prices, because even in negative reviews, there is still less dissatisfaction due to the price factor. However, the utility satisfaction degree of the low price strategy for consumers is showing a trend of diminishing returns, so it is more necessary for pharmaceutical e-commerce websites and enterprises to think out more sales strategies to please their consumers.

By contrast, customers pay more attention to the service quality; as shown in Figure 4g, the prevalence of pre-sales consulting in positive reviews is increasing over time as well as being essentially unchanged in negative reviews. As we described earlier, this again demonstrates customers’ growing focus on services. Pharmaceutical e-commerce websites and enterprises have improved the service level of pre-sales consulting, and this effort has been delivered to customers in a timely and effective manner, further forming part of their satisfaction.

Figure 4l shows that although the overall customer attention obtained by the package (Topic 1) is not high, the topic prevalence increases significantly in positive reviews and decreases significantly in negative ones. This indicates that the improvement of the comprehensive level of the drug packaging made by manufacturers has a significant effect, and this factor will gain extra favorable reviews on the premise that the drug meets the psychological expectations of customers.

## 6. Discussion and Conclusions

Our study aims to analyze what concerns consumers when they buy drugs online by using online reviews. To our knowledge, our study is the first to apply the STM model to online drug reviews. Compared with the existing literature methods, the STM model has obvious advantages in finding and measuring consumer concerns including satisfaction and dissatisfaction. Firstly, the original research method based on questionnaires was not only laborious and difficult to use to obtain a large number of data samples, but also had to rely on high-level experts to define a set of standard variables in advance. Our method not only can obtain a large number of samples at low cost but also relaxes the requirements of predefined variables and expert level. We embody the user-centric management philosophy by paying more attention to online user reviews. At the same time, we demonstrate the applicability of the topic model to drug reviews as we captured new topics that were not defined in the original questionnaire. Secondly, our research uses a structured topic model that can more effectively measure these consumer concerns compared with the LDA wildly used in previous literature. The STM model has obvious advantages in that it can implement document-level covariates (the extremity of reviews in our study) into the prior distribution of document-topics and topic-words so that we can do richer research and explore the concerns associated with user dissatisfaction among many elements. Finally, we take full advantage of the advantages of online reviews as a data sample and analyze the changing trends of topics over time, which is not possible with the questionnaire method. 

Our study also provides important practical management implications for pharmaceutical e-commerce websites and enterprises. First of all, our research provides a basis for companies to improve services and design marketing strategies topics by using the STM to find 12 topics. More importantly, we find three topics that were almost not appearing in the original questionnaire, including the expiration date of drugs, after-sales service and product packaging, which enriches the enterprise decision database. Secondly, we add the extremity of reviews as a covariate to the document-topic prior distribution to capture the five negative topics, which are the true voice of customer complaints. This is because the five topics we identified are not what customers are talking about in negative reviews but statistically appear more frequently in negative reviews than in positive reviews. This comparison strategy helps researchers and drug-related companies reveal the true voice of customer dissatisfaction [26]. The expiration date of drugs and after-sales service are the two most important factors of dissatisfaction, which have a significant impact on the reputation of the merchant. However, these two topics are not found in previous literature, which highlights the significance of our research. Finally, we analyze the trends of topics over time. The topic prevalence of the five dissatisfaction factors in the negative reviews has no obvious downward trend over time, except for distribution services. It shows that pharmaceutical e-commerce websites and enterprises still have great room for improving consumer satisfaction. Among the seven satisfactory factors, the topic prevalence of positive reviews of price has shown a downward trend over time, which indicates that the low-price strategies implemented by pharmaceutical e-commerce websites and pharmaceutical enterprises are becoming less attractive to customers. On the contrary, customers pay more attention to the improvement of service quality. These findings provide a decision basis for the adjustment of business strategies.

Our study has a set of limitations that can be explored in future research. Firstly, we collect drug review data from just one platform. Future research could collect more review data of different categories of drugs from different platforms to better summarize the research results. Secondly, our research adds the extremity and time of reviews as covariates to the STM model, while future studies could incorporate more covariates. One of them is to include prescription and over-the-counter drugs as covariates in the study. Customers can buy both of them online currently, but the regulation of prescription medicines is stricter. In addition, the type of platform can also be added as a covariate in future research. The platform for online drug purchases includes not only large e-commerce platforms such as JD.com, but also some small online pharmacies. Customers may pay different attention to factors when purchasing drugs on different platforms. Third, online reviews, as a reflection of the true voice of customers, are wildly used in different research areas such as product/service improvement and sales forecasting. Our study pays attention to the valuable data resource of drug reviews for the first time. More research based on drug reviews in the future will enrich the literature on the management of pharmaceutical enterprises and the development of pharmaceutical e-commerce.

## Figures and Tables

**Table 1:** Topic summary.

#	Topic Labels	Topic Proportions	Top Words	Appeared in Literate
1	Package	2.04%	box, product packaging, glass bottle, cases, careful, big, liquids	NO
2	After-sales service	6.13%	consumer, solve, returns, business deception, quality, prescription drugs, advertising	NO
3	Price	6.81%	cost-effective, low, cheap, convenience, coupons, small, free-shipping	YES
4	Side effects	3.74%	instruction manual, side effects, diarrhea, poor effect, sore throat, garbage, outer packing	YES
5	Delivery	9.65%	logistics, slow, cost, home delivery, boxes, pills, reasonable	YES
6	Curative effect	11.30%	significant, desired effect, thumbs, breathability, elderly, fall, symptomatic	YES
7	Brand image	10.84%	habituation, time-honored, bar code, quality, commodity, regular customers, trustworthy	YES
8	Expiration date	12.60%	production date, shelf life, expiration date, expired, sent over, help, attitude	NO
9	Main functions	21.46%	mouth ulcers, the trots, sneezing, aerosol, health products, laryngitis, packaging	YES
10	Only online purchase	4.88%	physical stores, unavailable, online orders, essentials, necessities, bag, spray	YES
11	Mailing service	3.97%	express fees, staff, parcel, consignment, waste, stores, angina	YES
12	Pre-sales consulting	6.58%	customer service, consulting, waiter, thoughtful, unbearable, dependent, digestion	YES

## References

[1] CSIP, LegitScript The Internet Pharmacy Market in 2016. http://www.safemedsonline.org/wp-content/uploads/2016/01/The-Internet-Pharmacy-Market-in-2016.pdf.

[2] FDA Survey Highlights in 2013. http://www.fda.gov/Drugs/ResourcesForYou/Consumers/BuyingUsingMedicineSafely/BuyingMedicinesOvertheInternet/BeSafeRxKnowYourOnlinePharmacy/ucm318497.htm〉.

[3] Orizio G., Merla A., Schulz P.J., Gelatti U. (2011). Quality of Online Pharmacies and Websites Selling Prescription Drugs: A Systematic Review. J. Med. Internet Res..

[4] Bate R., Jin G.Z., Mathur A. (2013). In Whom We Trust: The Role of Certification Agencies in Online Drug Markets. BE. J. Econ. Anal. Policy.

[5] CIPA Survey of Americans in 2015. http://www.cipa.com/news/cipa-survey-of-americans-finds-64-purchase-medications-from-canada-due-to-affordability/#more-676〉.

[6] Abanmy N. (2017). The Extent of Use of Online Pharmacies in Saudi Arabia. Saudi Pharm. J..

[7] Monteith S., Glenn T. (2018). Searching Online to Buy Commonly Prescribed Psychiatric Drugs. Psychiatry Res..

[8] LaValle S., Lesser E., Shockley R., Hopkins M.S., Kruschwitz N. (2011). Big Data, Analytics and the Path from Insights to Value. MIT Sloan Manag. Rev..

[9] Levy S.E., Duan W., Boo S. (2013). An Analysis of One-Star Online Reviews and Responses in the Washington, DC, Lodging Market. Cornell Hosp. Q..

[10] Mankad S., Han H., Goh J., Gavirneni S. (2016). Understanding Online Hotel Reviews through Automated Text Analysis. Serv. Sci..

[11] Korfiatis N., Stamolampros P., Kourouthanassis P., Sagiadinos V. (2019). Measuring Service Quality from Unstructured Data: A Topic Modeling Application on Airline Passengers’ Online Reviews. Expert Syst. Appl..

[12] Tirunillai S., Tellis G.J. (2014). Mining Marketing Meaning from Online Chatter: Strategic Brand Analysis of Big Data Using Latent Dirichlet Allocation. J. Mark. Res..

[13] Xiao S., Wei C.-P., Dong M. (2016). Crowd Intelligence: Analyzing Online Product Reviews for Preference Measurement. Inf. Manag..

[14] Bi J.-W., Liu Y., Fan Z.-P., Zhang J. (2019). Wisdom of Crowds: Conducting Importance-Performance Analysis (IPA) Through Online Reviews. Tour. Manag..

[15] Hu N., Zhang T., Gao B., Bose I. (2019). What Do Hotel Customers Complain about? Text Analysis Using Structural Topic Model. Tour. Manag..

[16] Lowes R. (2000). Are Online Pharmacies Good for Your Patients—And for You?. Med. Econ..

[17] Gallagher J.C., Colaizzi J.L. (2000). Issues in Internet Pharmacy Practice. Ann. Pharmacother..

[18] Liang C., Gu D., Tao F., Jain H.K., Zhao Y., Ding B. (2017). Influence of Mechanism of Patient-Accessible Hospital Information System Implementation on Doctor—Patient Relationships: A Service Fairness Perspective. Inf. Manag..

[19] Gu D., Deng S., Zheng Q., Liang C., Wu J. (2019). Impacts of Case-Based Health Knowledge System in Hospital Management: The Mediating Role of Group Effectiveness. Inf. Manag..

[20] Anand A., Sethi N., Sharon G., Mathew G., Songara R., Kumar P. (2010). Internet Pharmacy: Need to Be Implemented in India. Chron. Young Sci..

[21] Woonsocket R.I., Minneapolis M.N. News Release in 2015. http://corporate.target.com/article/2015/12/cvs-target-acquisition-complete.

[22] Macias W., Lewis L.S. (2006). How Well Do Direct-to-Consumer (DTC) Prescription Drug Web Sites Meet FDA Guidelines and Public Policy Concerns?. Health Mark. Q..

[23] Mackey T.K., Liang B.A. (2013). Pharmaceutical Digital Marketing and Governance: Illicit Actors and Challenges to Global Patient Safety and Public Health. Glob. Health.

[24] Gelatti U., Pedrazzani R., Marcantoni C., Mascaretti S., Repice C., Filippucci L., Zerbini I., Dal Grande M., Orizio G., Feretti D. (2013). ‘You’ve Got M@ Il: Fluoxetine Coming Soon!’: Accessibility and Quality of a Prescription Drug Sold on the Web. Int. J. Drug Policy.

[25] Huang B., Xu M. (2017). Commentary: Combating Sale of Counterfeit and Falsified Medicines Online: A Losing Battle. Front. Pharmacol..

[26] Fung C.H., Woo H.E., Asch S.M. (2004). Controversies and Legal Issues of Prescribing and Dispensing Medications Using the Internet. Mayo Clinic Proceedings.

[27] Van Hout M.C., Bingham T. (2014). Responsible Vendors, Intelligent Consumers: Silk Road, the Online Revolution in Drug Trading. Int. J. Drug Policy.

[28] Rajagopal M. (2016). Internet Pharmacies—Boon or Threat?. Prog. Neurol. Psychiatry.

[29] Chordiya S.V., Garge B.M. (2019). E-Pharmacy vs Conventional Pharmacy. IJCAAP.

[30] Fittler A., Vida R.G., Káplár M., Botz L. (2018). Consumers Turning to the Internet Pharmacy Market: Cross-Sectional Study on the Frequency and Attitudes of Hungarian Patients Purchasing Medications Online. J. Med. Internet Res..

[31] Sugiura L. (2018). Opportunities in Online Medicine Purchasing. Respectable Deviance and Purchasing Medicine Online.

[32] Poulas K., Avgerinou P. The online Pharmacy Market in Greece in 2018. https://www.researchgate.net/publication/323837284_The_online_pharmacy_market_in_Greece.

[33] Abu-Heija A.A., Shatta M., Ajam M., Abu-Heija U., Imran N., Levine D. (2019). Quantitative Readability Assessment of the Internal Medicine Online Patient Information on Annals. Org. Cureus.

[34] Jacobi C., Van Atteveldt W., Welbers K. (2016). Quantitative Analysis of Large Amounts of Journalistic Texts Using Topic Modelling. Digit. J..

[35] Jiao J.R., Simpson T.W., Siddique Z. (2007). Product Family Design and Platform-Based Product Development: A State-of-the-Art Review. J. Intell. Manuf..

[36] Zhu F., Zhang X. (2010). Impact of Online Consumer Reviews on Sales: The Moderating Role of Product and Consumer Characteristics. J. Mark..

[37] Groves R.M. (2006). Nonresponse Rates and Nonresponse Bias in Household Surveys. Public Opin. Q..

[38] Banerjee S., Chua A.Y. (2016). In Search of Patterns among Travellers’ Hotel Ratings in TripAdvisor. Tour. Manag..

[39] Boo S., Busser J.A. (2018). Meeting Planners’ Online Reviews of Destination Hotels: A Twofold Content Analysis Approach. Tour. Manag..

[40] Duan W., Yu Y., Cao Q., Levy S. (2016). Exploring the Impact of Social Media on Hotel Service Performance: A Sentimental Analysis Approach. Cornell Hosp. Q..

[41] Guo Y., Barnes S.J., Jia Q. (2017). Mining Meaning from Online Ratings and Reviews: Tourist Satisfaction Analysis Using Latent Dirichlet Allocation. Tour. Manag..

[42] Gebauer J., Tang Y., Baimai C. (2008). User Requirements of Mobile Technology: Results from a Content Analysis of User Reviews. Inf. Syst. e-Bus. Manag..

[43] Xu B., Liang J., Xie C., Liang B., Chen L., Xiao Y. (2019). CN-DBpedia2: An Extraction and Verification Framework for Enriching Chinese Encyclopedia Knowledge Base. Data Intell..

[44] Salatino A.A., Thanapalasingam T., Mannocci A., Birukou A., Osborne F., Motta E. (2019). The Computer Science Ontology: A Comprehensive Automatically-Generated Taxonomy of Research Areas. Data Intell..

[45] Jeong B., Yoon J., Lee J.-M. (2019). Social Media Mining for Product Planning: A Product Opportunity Mining Approach Based on Topic Modeling and Sentiment Analysis. Int. J. Inf. Manag..

[46] Ko N., Jeong B., Choi S., Yoon J. (2017). Identifying Product Opportunities Using Social Media Mining: Application of Topic Modeling and Chance Discovery Theory. IEEE Access.

[47] Ibrahim N.F., Wang X. (2019). A Text Analytics Approach for Online Retailing Service Improvement: Evidence from Twitter. Decis. Support Syst..

[48] Blei D.M., Ng A.Y., Jordan M.I. (2003). Latent Dirichlet Allocation. J. Mach. Learn. Res..

[49] Chen D., Zhang R., Liu K., Hou L. (2018). Knowledge Discovery from Posts in Online Health Communities Using Unified Medical Language System. Int. J. Environ. Res. Public Health.

[50] Zhao Y., Zhang J., Wu M. (2019). Finding Users’ Voice on Social Media: An Investigation of Online Support Groups for Autism-Affected Users on Facebook. Int. J. Environ. Res. Public Health.

[51] Xiao C., Zhang P., Chaovalitwongse W.A., Hu J., Wang F. Adverse Drug Reaction Prediction with Symbolic Latent Dirichlet Allocation. Proceedings of the Thirty-First AAAI Conference on Artificial Intelligence.

[52] Zhang Y., Chen M., Huang D., Wu D., Li Y. (2017). IDoctor: Personalized and Professionalized Medical Recommendations Based on Hybrid Matrix Factorization. Future Gener. Comput. Syst..

[53] Paul M.J., Dredze M. You Are What You Tweet: Analyzing Twitter for Public Health. Proceedings of theFifth International AAAI Conference on Weblogs and Social Media.

[54] James T.L., Calderon E.D.V., Cook D.F. (2017). Exploring Patient Perceptions of Healthcare Service Quality through Analysis of Unstructured Feedback. Expert Syst. Appl..

[55] Botsis T., Buttolph T., Nguyen M.D., Winiecki S., Woo E.J., Ball R. (2012). Vaccine Adverse Event Text Mining System for Extracting Features from Vaccine Safety Reports. J. Am. Med. Inform. Assoc..

[56] Hao H., Zhang K. (2016). The Voice of Chinese Health Consumers: A Text Mining Approach to Web-Based Physician Reviews. J. Med. Internet Res..

[57] Roberts M.E., Stewart B.M., Tingley D., Lucas C., Leder-Luis J., Gadarian S.K., Albertson B., Rand D.G. (2014). Structural Topic Models for Open-Ended Survey Responses. Am. J. Political Sci..

[58] Liu Y., Bi J.-W., Fan Z.-P. (2017). A Method for Ranking Products through Online Reviews Based on Sentiment Classification and Interval-Valued Intuitionistic Fuzzy TOPSIS. Int. J. Inf. Technol. Decis. Mak..

[59] Kuhn K.D. (2018). Using Structural Topic Modeling to Identify Latent Topics and Trends in Aviation Incident Reports. Transp. Res. Part C Emerg. Technol..

[60] Blei D.M. (2012). Probabilistic Topic Models. Commun. ACM.

[61] Sun X., Han M., Feng J. (2019). Helpfulness of Online Reviews: Examining Review Informativeness and Classification Thresholds by Search Products and Experience Products. Decis. Support Syst..

[62] Qi J., Zhang Z., Jeon S., Zhou Y. (2016). Mining Customer Requirements from Online Reviews: A Product Improvement Perspective. Inf. Manag..

[63] Jing N., Jiang T., Du J., Sugumaran V. (2018). Personalized Recommendation Based on Customer Preference Mining and Sentiment Assessment from a Chinese E-Commerce Website. Electron. Commer. Res..

[64] Hu N., Pavlou P.A., Zhang J.J. (2017). On Self-Selection Biases in Online Product Reviews. MIS Q..

[65] Gao G.G., Greenwood B.N., Agarwal R., McCullough J. (2015). Vocal Minority and Silent Majority: How Do Online Ratings Reflect Population Perceptions of Quality?. MIS Q..

[66] Lu S.F., Rui H. (2018). Can We Trust Online Physician Ratings? Evidence from Cardiac Surgeons in Florida. Manag. Sci..

[67] Hu N., Zhang J., Pavlou P.A. (2009). Overcoming the J-Shaped Distribution of Product Reviews. Commun. ACM.

[68] Roberts M.E., Stewart B.M., Tingley D. (2017). Stm: R Package for Structural Topic Models. r Package Version 1.3. 3. J. Stat. Softw. (Forthcom.) Lat. Conf. Latinoam. Sobre Uso R Investig. Desarro..

[69] Iacus S.M., King G., Porro G. (2012). Causal Inference without Balance Checking: Coarsened Exact Matching. Political Anal..

[70] Huang L., Dou Z., Hu Y., Huang R. (2019). Textual Analysis for Online Reviews: A Polymerization Topic Sentiment Model. IEEE Access..

[71] Roberts M.E., Stewart B.M., Airoldi E.M. (2016). A Model of Text for Experimentation in the Social Sciences. J. Am. Stat. Assoc..

[72] Sutherland I., Sim Y., Lee S.K., Byun J., Kiatkawsin K. (2020). Topic Modeling of Online Accommodation Reviews via Latent Dirichlet Allocation. Sustainability.

[73] Papathanassis A., Knolle F. (2011). Exploring the Adoption and Processing of Online Holiday Reviews: A Grounded Theory Approach. Tour. Manag..

[74] Yin D., Mitra S., Zhang H. (2016). Research Note—When Do Consumers Value Positive vs. Negative Reviews? An Empirical Investigation of Confirmation Bias in Online Word of Mouth. Inf. Syst. Res..

[75] Wu J., Wang Y., Zhang R., Cai J. (2018). An Approach to Discovering Product/Service Improvement Priorities: Using Dynamic Importance-Performance Analysis. Sustainability.

[76] Pizam A., Shapoval V., Ellis T. (2016). Customer Satisfaction and Its Measurement in Hospitality Enterprises: A Revisit and Update. Int. J. Contemp. Hosp. Manag..

